# Supplementary material for: Hippocampal firing fields anchored to a moving object predict homing direction during path-integration-based behavior
Source: Nat Commun. 2023 Nov 15;14:7373. doi: 10.1038/s41467-023-42642-3 (PMC10651862; doi:10.1038/s41467-023-42642-3)
Supplement: Supplementary file 1 — Supplementary Information [file 41467_2023_42642_MOESM1_ESM.pdf]

Supplementary Information

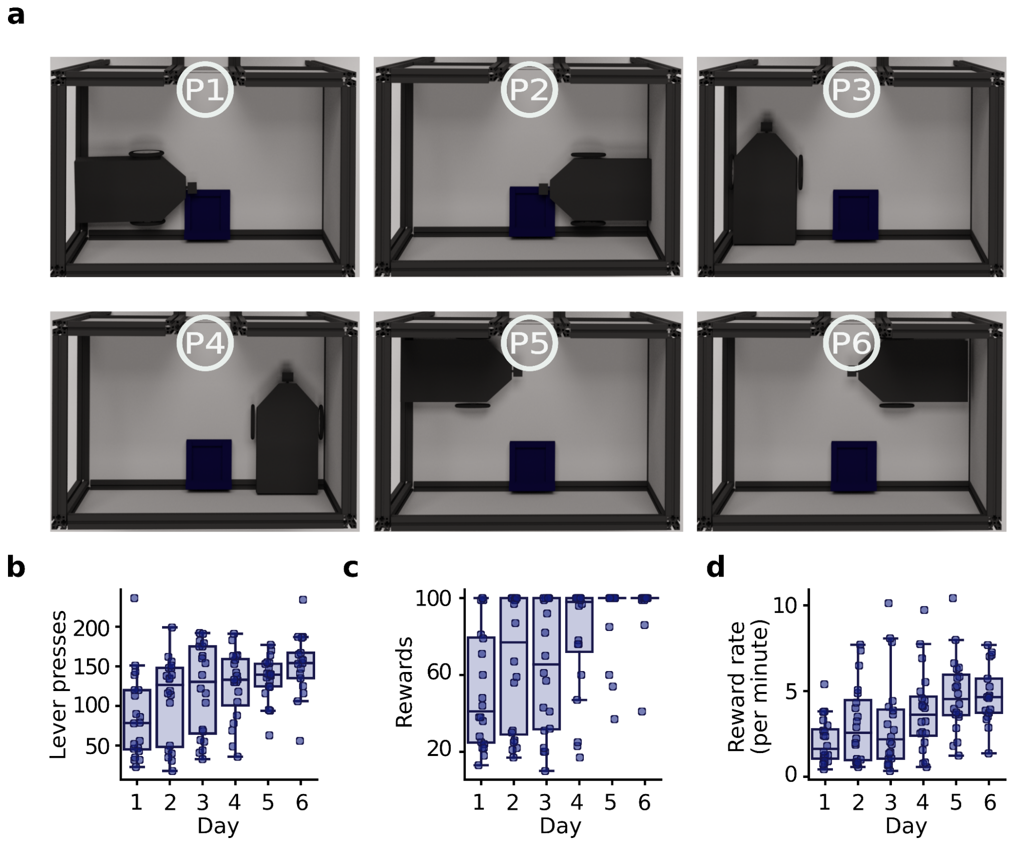

Supplementary Fig. 1: **Mice learned to press the lever in the home base**. **a**, Schematic showing the different positions of the lever within the home base when the mice learned to press the lever to trigger food delivery in the food magazine. The lever changed position between days (from P1 to P6). **b**, **c**, and **d**: Number of lever presses, rewards delivered, and reward rate across six training days (P1 to P6) with the lever inside the home base (n = 13 mice). Source data are provided as a Source Data file.

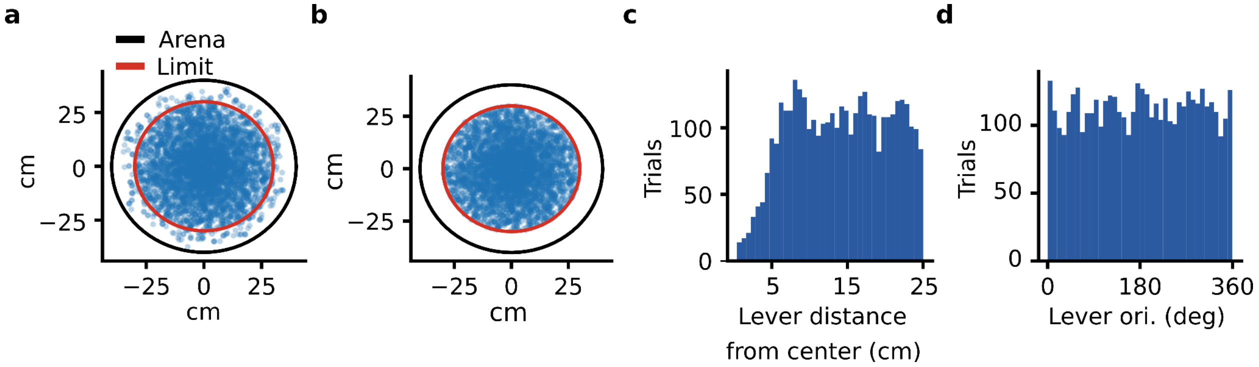

Supplementary Fig. 2: **Position and orientation of the lever box on the arena. a**, Distribution of lever box position on the arena for trials of the test sessions. The red circle indicated the maximal distance (25 cm) between the lever box center and the arena center for a trial to be included in the analysis. **b**, The lever box position distribution for trials included in the analysis. **c**, Distribution of distance between the lever box and the center of the arena for all trials of the test sessions. **d**, Distribution of lever box orientation for all trials of the test sessions. Source data are provided as a Source Data file.

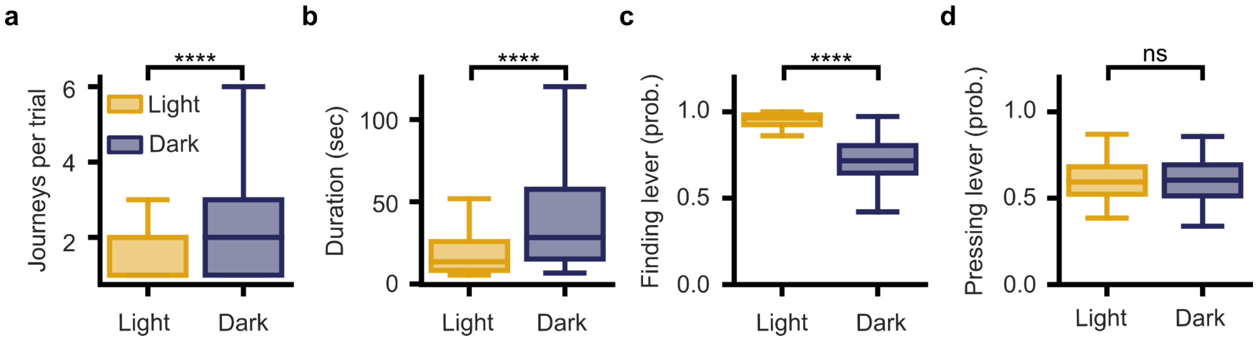

Supplementary Fig. 3: **Behavioral characteristics of light and dark trials.** The plots show the distribution of trials or recording sessions, but statistical significance was established using mice as statistical units. The analysis is similar to that presented in **Fig. 1h-k**. **a**, Number of journeys per trial for light and dark trials (2429 light and 2024 dark trials, stats: n = 13 mice, two-sided Wilcoxon signed-rank test, $P = 2.44 \times 10^{-4}$). **b**, Trial duration for light and dark trials (stats: n = 13 mice, two-sided Wilcoxon signed-rank test, $P = 2.44 \times 10^{-4}$). **c**, Probability of finding the lever on journeys associated with light and dark trials (n = 67 sessions, stats: n = 13 mice, two-sided Wilcoxon signed-rank test, $P = 2.44 \times 10^{-4}$). **d**, Probability of pressing the lever once the lever had been found during light and dark trials (n = 67 sessions, stats: n = 13 mice, two-sided Wilcoxon signed-rank test, $P = 0.78$). ***$P < 0.001$,ns: non-significant. Source data are provided as a Source Data file.

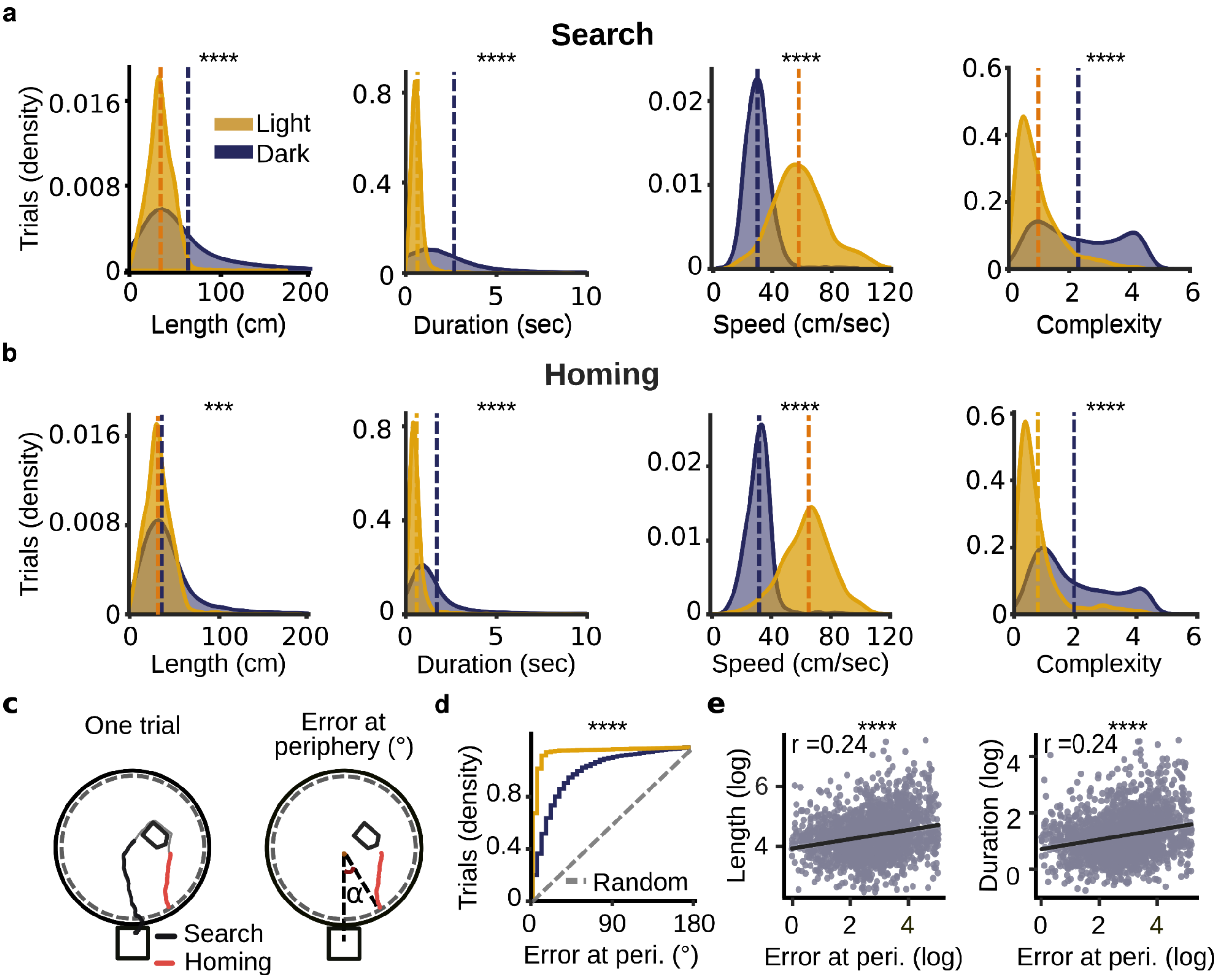

Supplementary Fig. 4: Characteristics of search and homing paths during light and dark trials. The plots from **a** and **b** show the distribution of trials, but statistical significance was established using mice as statistical units (as in **Fig2. b-c**). **a**, Distribution of search path length during light and dark trials (n = 2429 light and 2024 dark trials, stats: n = 13 mice, two-sided Wilcoxon signed-rank test, $P = 2.4 \times 10^{-4}$), duration (stats: n = 13 mice, two-sided Wilcoxon signed-rank test, $P = 2.4 \times 10^{-4}$), speed (stats: n = 13 mice, two-sided Wilcoxon signed-rank test, $P = 2.4 \times 10^{-4}$), and complexity (stats: n = 13 mice, two-sided Wilcoxon signed-rank test, $P = 2.4 \times 10^{-4}$) for light and dark trials. The vertical dashed lines indicate the medians of the distributions (blue: dark trials, yellow: light trials). **b**, Distribution of homing path length (n = 2429 light and 2024 dark trials, stats: n = 13 mice, two-sided Wilcoxon signed-rank test, $P = 2.4 \times 10^{-4}$) , duration (n = 13 mice, two-sided Wilcoxon signed-rank test, $P = 2.4 \times 10^{-4}$), speed (n = 13 mice, two-sided two-sided Wilcoxon signed-rank test, $P = 2.4 \times 10^{-4}$), and complexity (n = 13 mice, two-sided Wilcoxon signed-rank test, $P = 2.4 \times 10^{-4}$) for light and dark trials. **c**, Left: Example of one trial in the AutoPI task. The search and homing paths are shown in black and red, respectively. Right: Schematic illustrating how error at the periphery was calculated. Error at the periphery is the angle between two vectors with the center of the arena as the origin. The first vector pointed toward the center of the bridge and the second vector pointed toward the position at which the mouse first reached the periphery of the arena. **d**, Cumulative distribution of homing error at periphery during light and dark trials. The trial distribution is shown, but statistical significance was established using mice as statistical units (stats: n = 13 mice, two-sided Wilcoxon signed-rank test, $P = 2.4 \times 10^{-4}$). The dashed line represents the distribution of error at the periphery expected by chance (homogeneous distribution). **e**, Relationship between homing error at the periphery and the search path length (n = 1963 dark trials; Pearson correlation; $P = 3.3 \times 10^{-26}$) and search path duration (n = 1963 dark trials; Pearson correlation; $P = 1.1 \times 10^{-26}$). The values were log-transformed ($ln(x + 1)$) to reduce the skewness of the distributions. The *r*-values refer to the

Pearson correlation coefficients. ***$P < 0.001$, ****$P < 0.0001$. Source data are provided as a Source Data file.

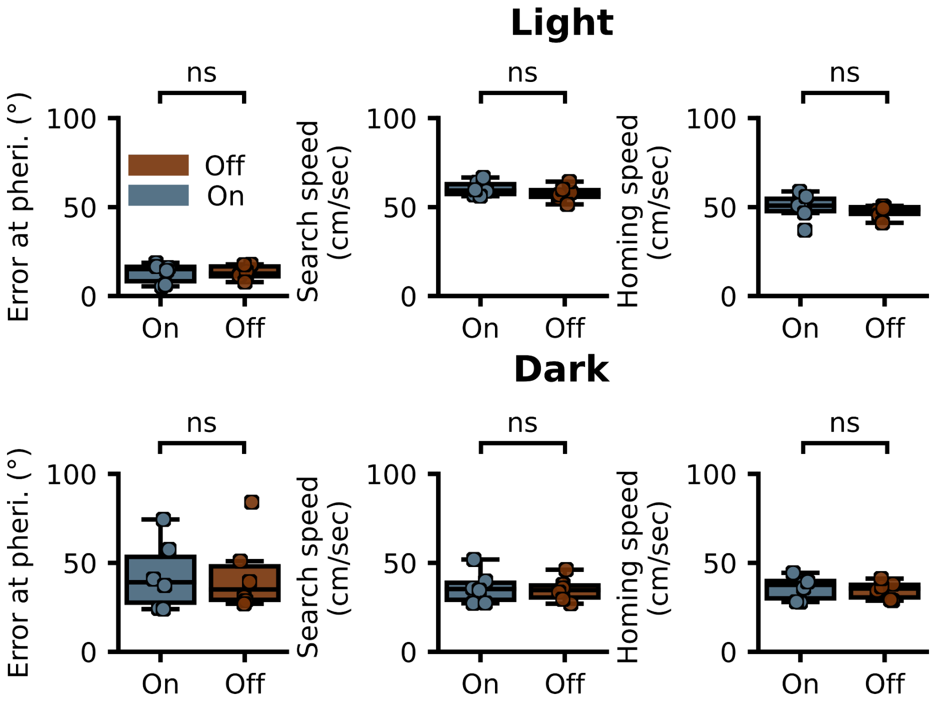

Supplementary Fig. 5: **Interfering with odor cues with an artificial airflow over the arena does not affect the performance on the AutoPI task.** Error at periphery and running speed during search and homing for light (top row) and dark (bottom row) trials with or without airflow (On: with airflow; Off: without airflow). There was no significant effect of airflow on homing error or running speed (n = 6 mice, Wilcoxon two-sided signed-rank test, Light trials: Error at periphery, P = 0.6; Search speed, P = 0.12; Homing speed, P = 0.12; Dark trials: Error at periphery, P = 0.25; Search speed, P = 0.37; Homing speed, P = 0.37). ns: non-significant. Source data are provided as a Source Data file.

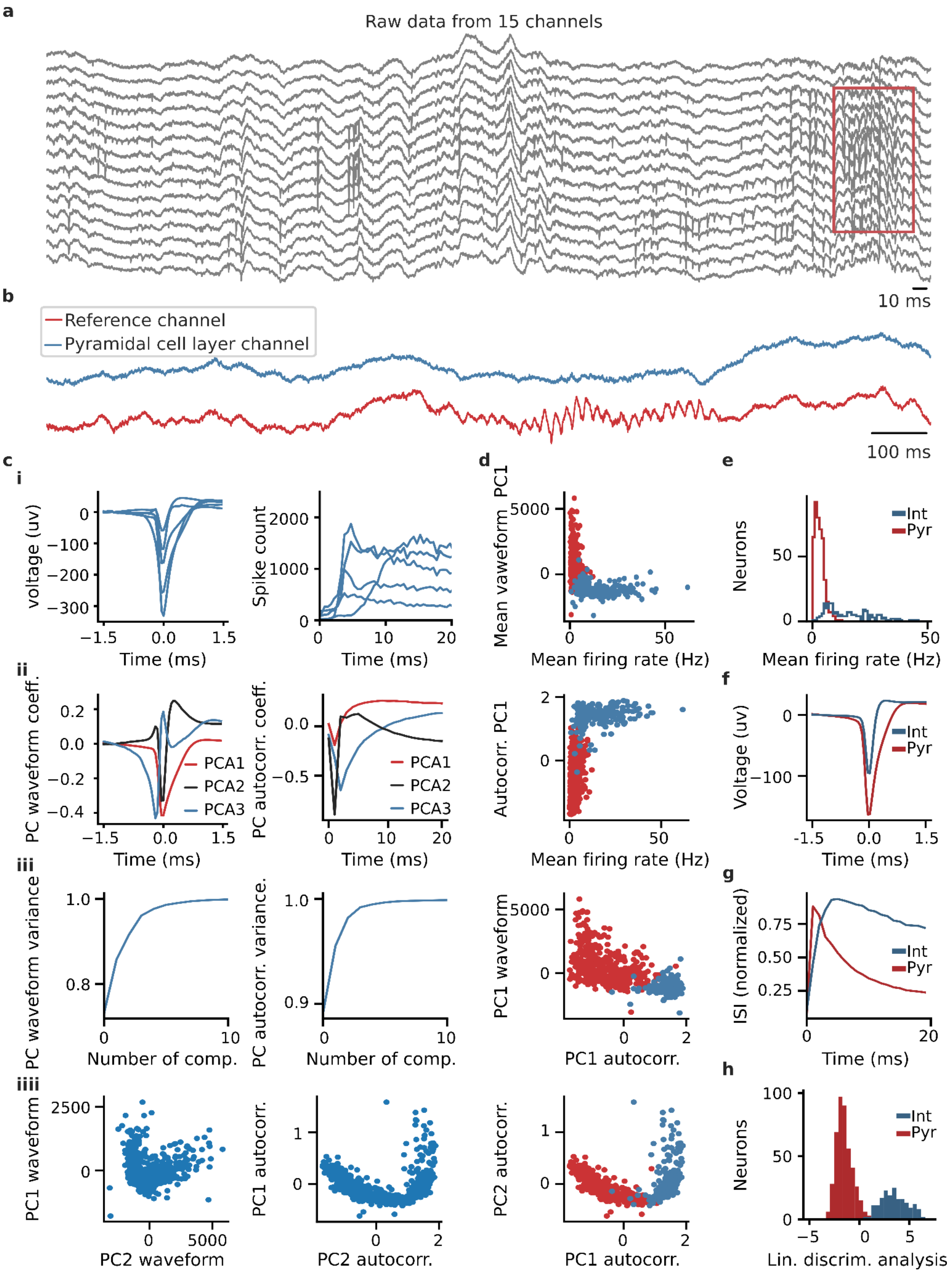

Supplementary Fig. 6: **Classification of recorded neurons into pyramidal cells and interneurons.** **a**, Examples of raw signals recorded from the CA1 region. The red rectangle highlights ripples recorded from the pyramidal cell layer. Only neurons recorded from probe shanks with ripples were included in the analysis. **b**, Examples of signals recorded near the pyramidal cell layer and further away (reference channel). **c**, Transformation of spike waveforms (left) and spike-time autocorrelations (right) using principal component analysis. **i**, Examples of spike waveforms and spike-time autocorrelations of neurons recorded in the pyramidal cell layer. **ii**, Coefficients of the first three principal components. **iii**, Proportion of the variance explained by the first ten components. **iiii**, Mean waveforms and spike-time

autocorrelations plotted in the first and second dimensions of the PCA. **d**, Results of a k-means clustering algorithm applied on the first three principal components of the waveforms, the first three principal components of the spike time autocorrelations, and the normalized firing rates. Neurons are plotted using different combinations of principal components and mean firing rate. **e**, Distribution of mean firing rate for putative pyramidal cells (Pyr) and interneurons (Int). **f**, Mean waveforms of pyramidal cells and interneurons. **g**, Mean spike-time autocorrelations of pyramidal cells and interneurons. **h**, Projection of a linear discriminant analysis (LDA) showing that the putative pyramidal cells and interneurons were largely non-overlapping populations. Source data are provided as a Source Data file.

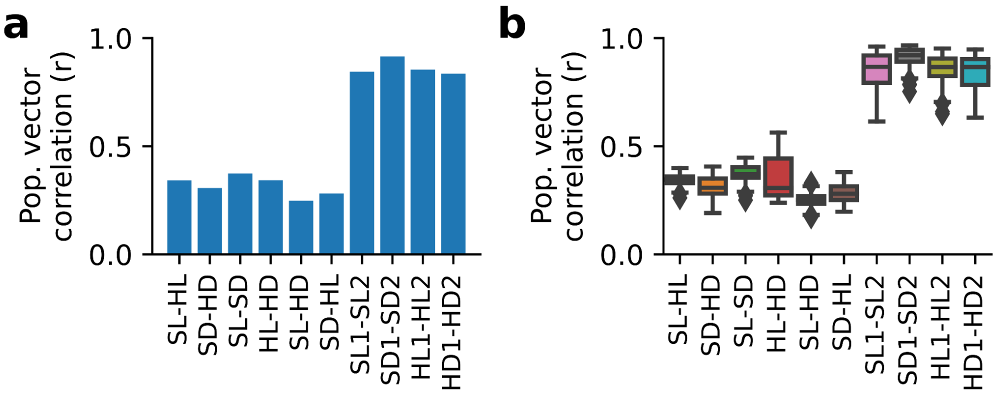

Supplementary Fig. 7: **Remapping in the zone of interest on the arena quantified by population vector analysis. a**, We created two 3D stacks of firing rate maps for each comparison including all pyramidal cells. The correlation coefficients were obtained by flattening the 3D stacks to 1D arrays and performing one Pearson correlation per comparison. **b**, We created two 3D stacks of firing rate maps for each comparison. A Pearson correlation was performed at each spatial bin of the firing rate maps (4x8, 5x5 cm bins). The boxplot shows the distribution of r values (from 32 spatial bins) when comparing different behavioral conditions (SL: Search-Light, HL: Homing-Light, SD: Search-Dark, HD: Homing-Dark). The trials of each condition were divided into two independent sets of trials (e.g., SL1 and SL2) to allow within-condition comparisons. Correlations between different conditions (e.g., SL-HL) were all significantly lower than those within conditions (e.g., SL1-SL2) (n = 32 spatial bins per comparison, two-sided Mann-Whitney U test, all $P = 6.5 \times 10^{-43}$); Source data are provided as a Source Data file.

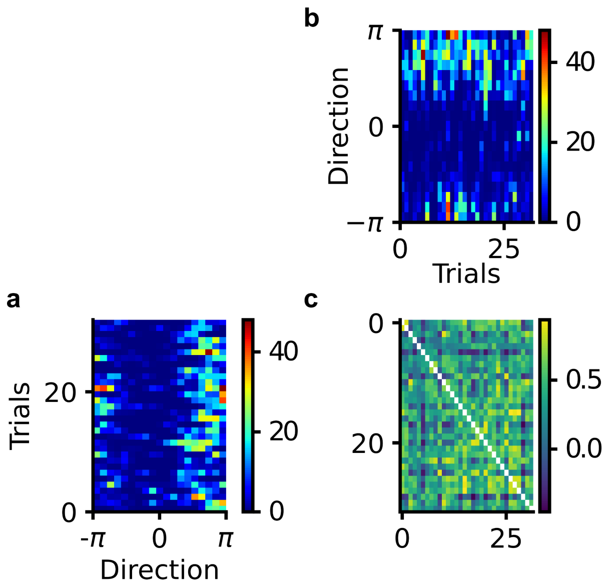

Supplementary Fig. 8: **Procedure to calculate the trial matrix correlation. a**, Trial matrix showing the firing rate of a neuron on each trial as a function of a behavioral variable, here the direction of the mouse around the lever box. **b**, Same trial matrix as in **a** but rotated by 90° anti-clockwise. **c**, Correlation matrix showing the Pearson correlation coefficients between all pairs of trials. Pairs of trials with similar firing rate vectors result from values approaching 1. The trial matrix correlation is the mean of the correlation matrix after excluding the values of the main diagonal. Source data are provided as a Source Data file.

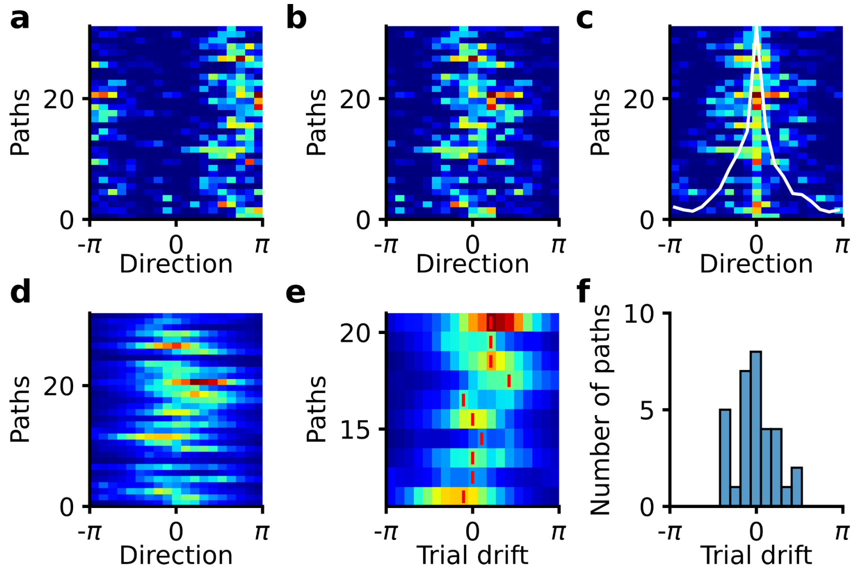

Supplementary Fig. 9: **Procedure to calculate the directional trial drift of lever-box-anchored fields.**

The directional trial drift is based on a cross-correlation between the single-trial firing rate of a neuron as a function of the direction around the lever box and an idealized tuning curve of the neuron. **a**, Trial matrix showing the firing rate of a neuron on every trial as a function of the direction of the mouse relative to the lever box. **b**, Trial matrix after shifting all trials together so that the highest mean firing rate is aligned to 0. This preserves the relative direction of firing across trials. **c**, Trial matrix after aligning the firing rate on each trial so that its peak is at 0. This eliminates the potential drift of the preferred direction on each trial. We calculated the mean firing rate as a function of direction (white line), which served as an idealized tuning curve of the neuron. **d**, Cross-correlation between the firing rate on each trial (matrix shown in **b**) and the idealized tuning curve of the neuron (white line in **c**). The cross-correlation between the idealized tuning curve and the firing rate estimate on single trials can be used to identify the shift of the tuning curve on single neurons. Compared to the single-trial firing rates (**b**), the cross-correlation (**d**) is smoother and provides a less noisy estimate of the trial drift. **e**, Same as in **d**, but fewer trials are shown. The vertical red bars, which are aligned to the peak of each trial's cross-correlation, represent the neuron's directional trial drift. **f**, Distribution of directional trial drift for a lever-box-anchored neuron during the dark trials of a single session. Source data are provided as a Source Data file.

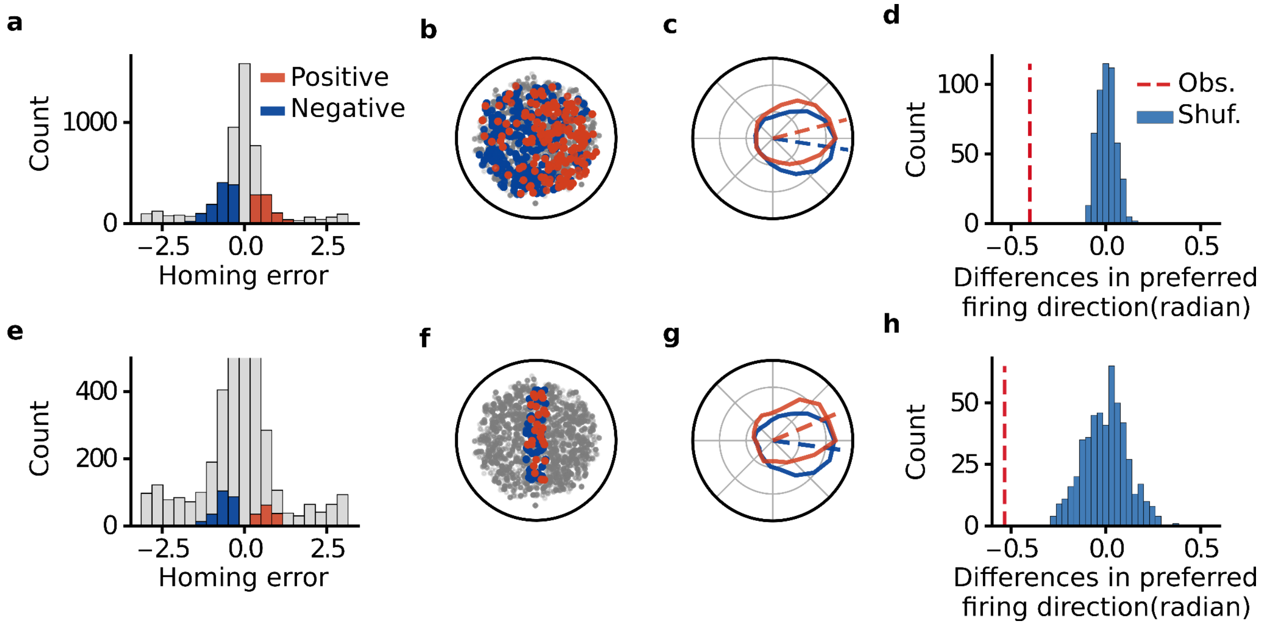

Supplementary Fig. 10: **Change in firing direction associated with the homing direction does not depend on lever position. a**, We selected dark trials with negative and positive errors at the periphery. The angle was calculated relative to a vector originating at the center of the lever box and pointed toward the bridge. Trials with errors larger than -π/2 and smaller than -π/10 are shown in blue, and trials with errors larger than π/10 and smaller than π/2 are shown in orange. **b**, Distribution of lever position for selected trials with negative (blue) and positive (red) error at the periphery. **c**, Mean firing direction of all neurons with lever-box-anchored fields (*n* = 109). For each neuron, the trial matrix was rotated along the x-axis until the highest firing rate of the neuron was at 0 radians. The trial matrices of the 108 neurons were combined, and trial matrices for the trials with negative and positive errors were generated. The polar plot shows the mean firing rate as a function of the direction obtained from the two matrices. **d**, Observed difference in preferred firing direction for trials with negative and positive error at the periphery (red dotted line) and distribution of the differences expected by chance (blue histogram). The chance distribution was obtained by shuffling the identity of trials with negative and positive error and calculating the difference in the preferred direction 500 times. The differences were larger than those observed by chance (n = 109, Pearson correlation, $P = 6.9 \times 10^{-5}$). **e** to **h**, Same as for **a** to **d**, but the analysis was limited to trials in which the lever box was located 5 cm from the center of the arena on the x-axis. Source data are provided as a Source Data file.

Examples from Fig3.b

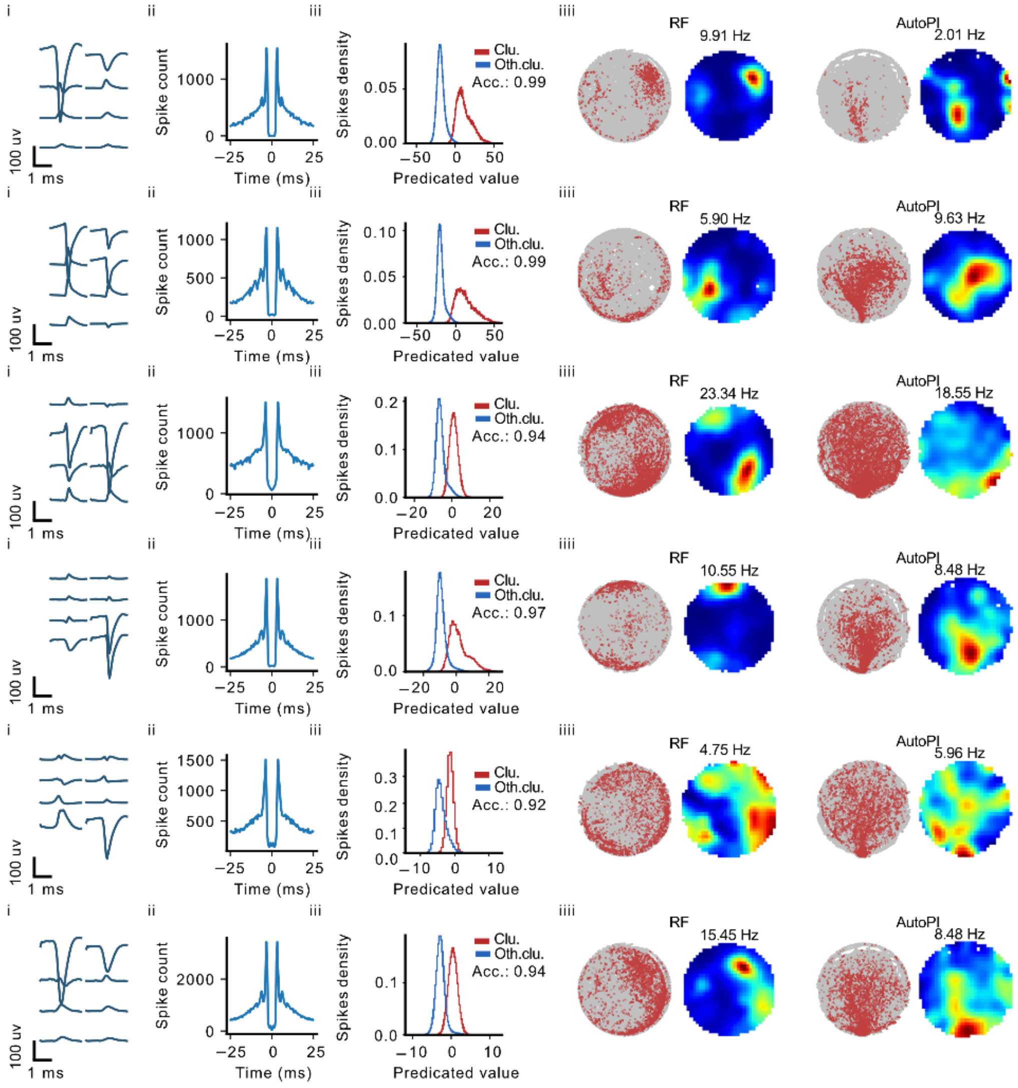

Examples from Fig3.e

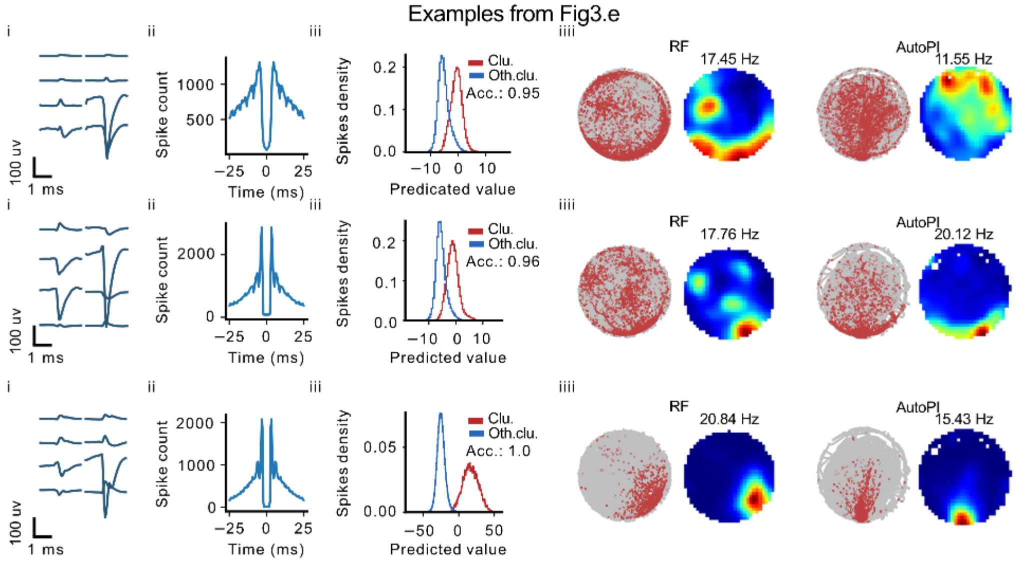

Part 1 from 4

Examples from Fig3.e

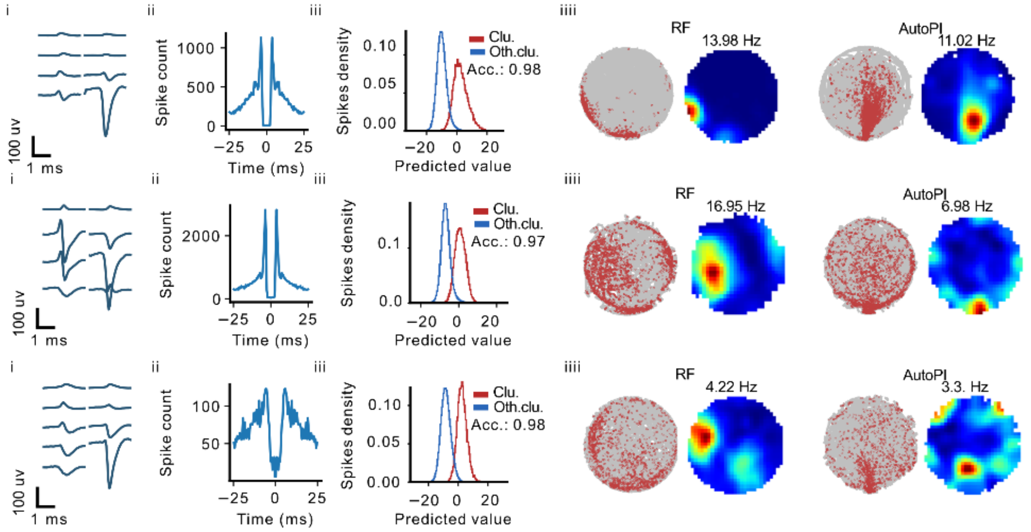

Examples from Fig4.a

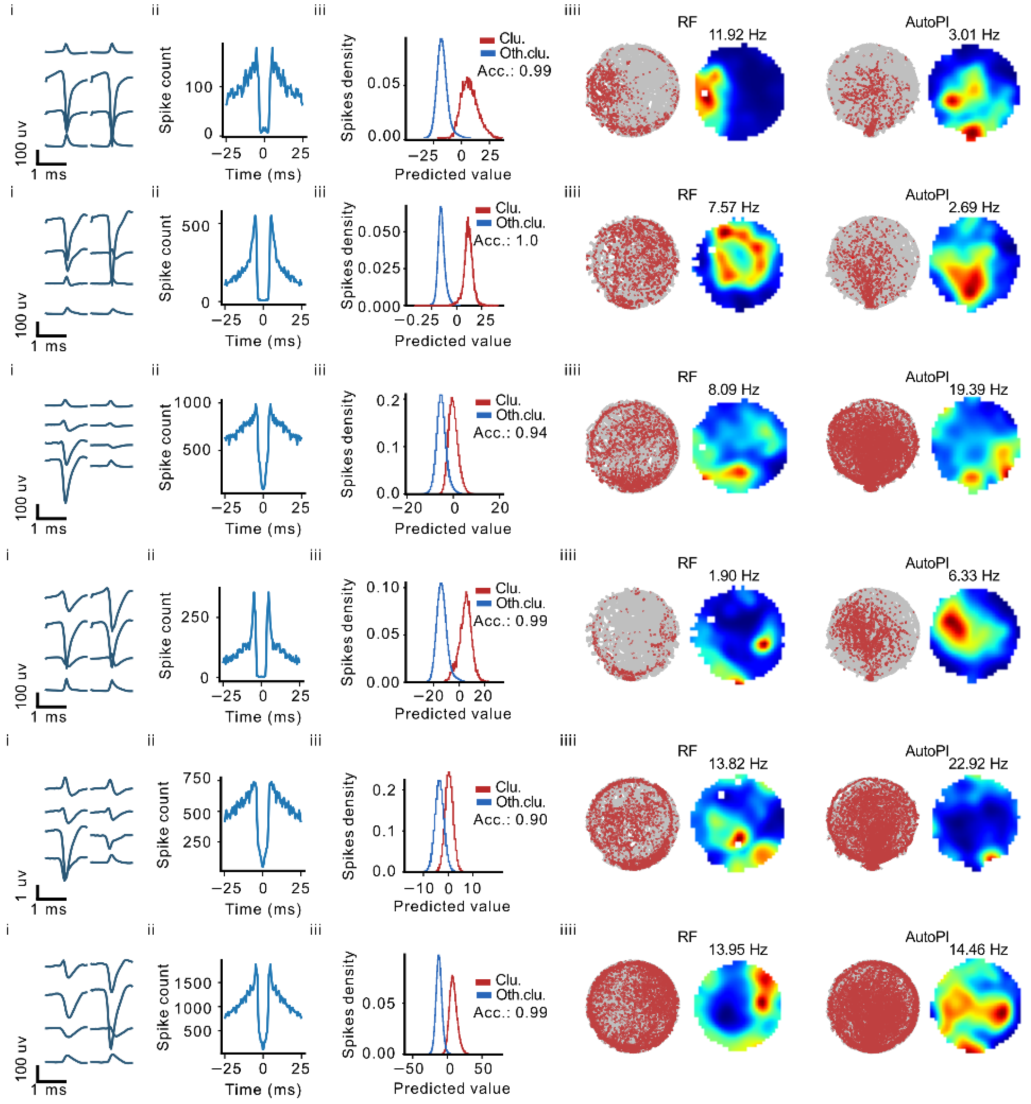

Part 2 from 4

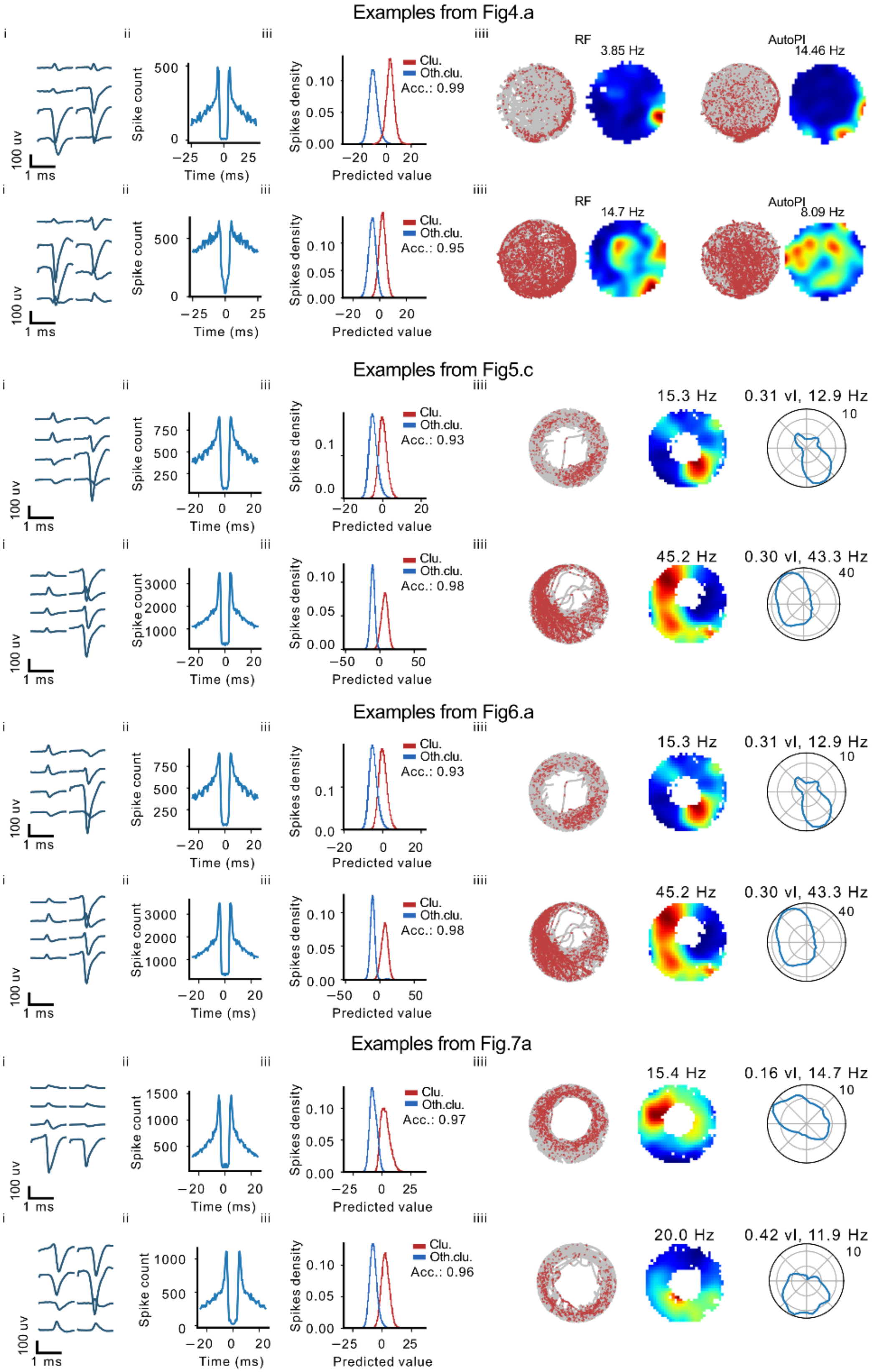
Examples from Fig4.a
i
ii
iii
iiii
RF
3.85 Hz
AutoPI
14.46 Hz
Spike count
Time (ms)
Spikes density
Predicted value
Clu.
Oth.clu.
Acc.: 0.99
100 uv
1 ms
RF
14.7 Hz
AutoPI
8.09 Hz
Acc.: 0.95
Examples from Fig5.c
15.3 Hz
0.31 vl, 12.9 Hz
Acc.: 0.93
45.2 Hz
0.30 vl, 43.3 Hz
Acc.: 0.98
Examples from Fig6.a
15.3 Hz
0.31 vl, 12.9 Hz
Acc.: 0.93
45.2 Hz
0.30 vl, 43.3 Hz
Acc.: 0.98
Examples from Fig.7a
15.4 Hz
0.16 vl, 14.7 Hz
Acc.: 0.97
20.0 Hz
0.42 vl, 11.9 Hz
Acc.: 0.96

Part 3 from 4

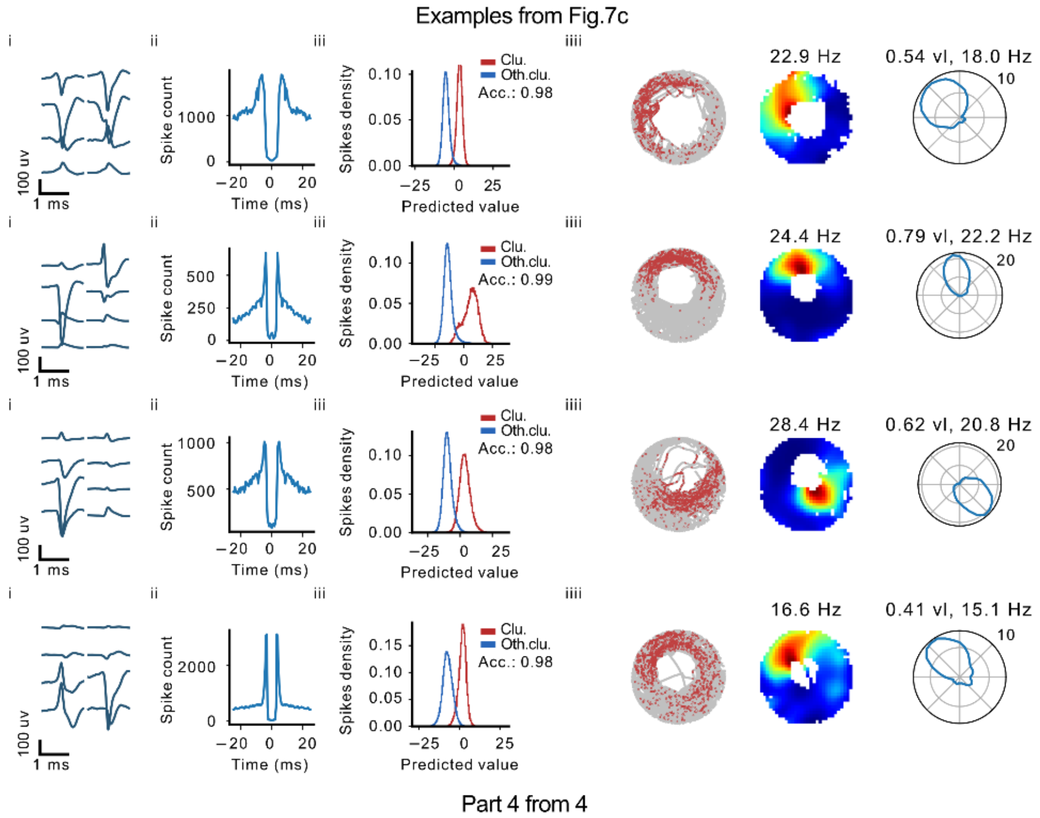

Supplementary Fig. 11: **In-depth information on example neurons of the main figures (Fig. 3b** and **three cells from Fig.3.e). i**. The mean waveform of the neuron on an 8-channel shank. **ii**. Spike-time autocorrelation of the neuron. **iii**. Results of a linear discrimination analysis (LDA) of the spike of the neurons (Clu) and those of other neurons (Oth. clu.). Waveform features were used as inputs to the LDA. The accuracy of the LDA is shown for each neuron (Acc.). **iiii**. Spikes on the path of the animal together with the firing rate map of the same neuron. The number on top of the firing rate map is the peak of the firing rate. Source data are provided as a Source Data file.

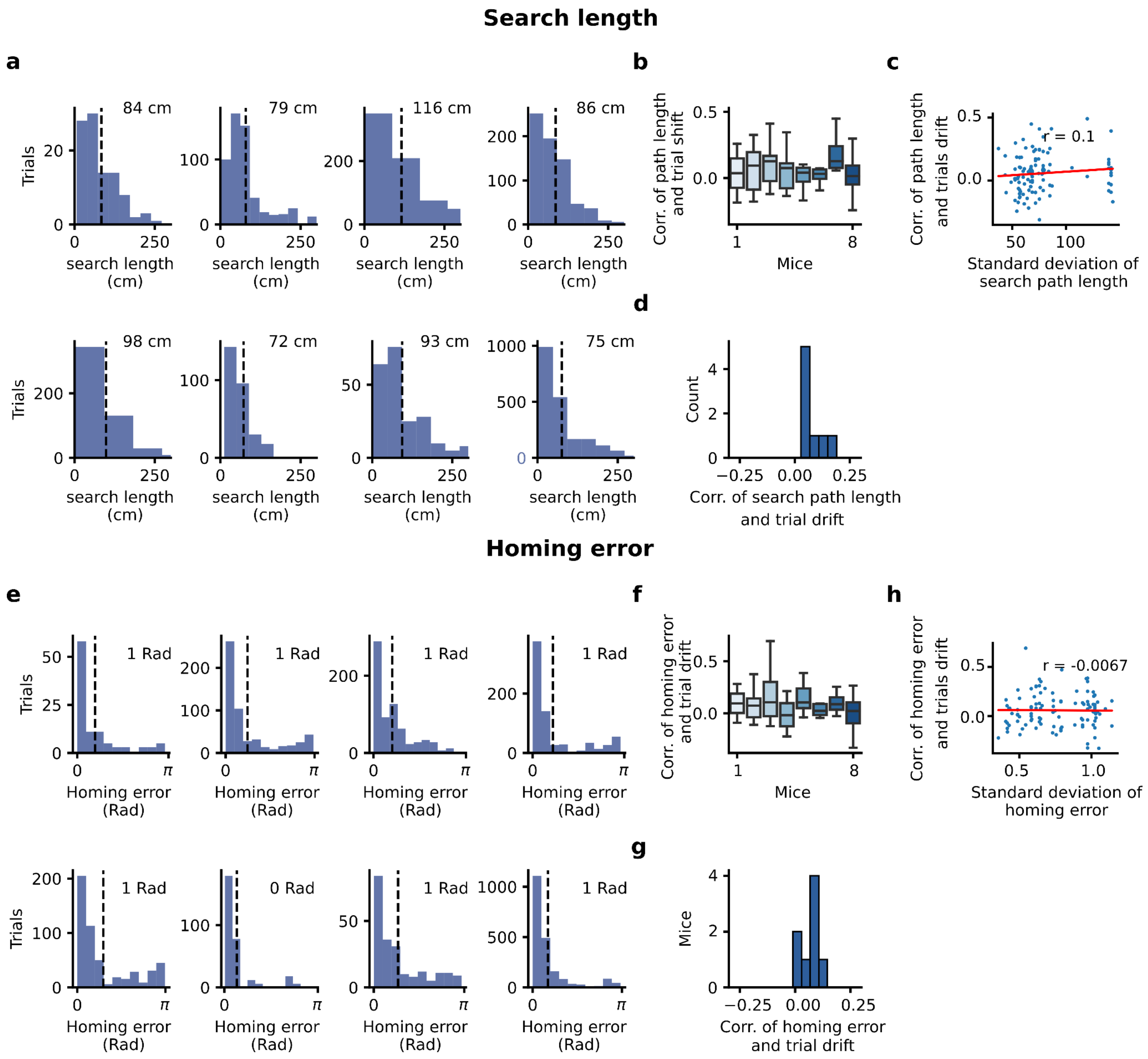

Supplementary Fig. 12. **a**, The distribution of search path lengths for eight mice. **b**, Distribution of Pearson correlation coefficients between the search path length and the trial drifts for each mouse. **c**, Distribution of median correlation between search path length and the trial drifts (n = 8, Pearson correlation, r = 0.1, P = 0.29). **d**, Distribution of Pearson correlation for the length of the search path and trial drift (n = 8, one-sided Wilcoxon signed-rank test, P = 0.0039). **e**, The distribution of homing error at periphery for eight mice. The dashed line represents the mean of the homing error for each mouse. **f**, Distribution of Pearson correlation coefficients between the homing error at periphery and the trial drift for each mouse. **g**, Distribution of Pearson correlation for the homing error at periphery and the trial drift (n = 8, one-sided Wilcoxon signed-rank test, P = 0.0078). **h**, Absence of linear relationship between the homing-error/trial-drift correlation coefficients and the standard deviation of homing error for each

lever-anchor field (n = 8 mice, Pearson correlation, r = -0.0067, P = 0.94). Source data are provided as a Source Data file.

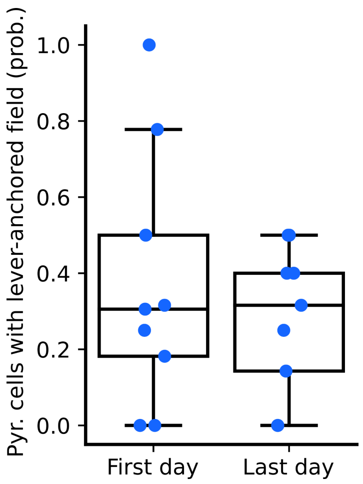

Supplementary Fig. 13: Proportion of pyramidal cells with lever-anchored fields during the first and last recording session of each mouse. There was no significant difference between the first and last recording day (n = 8, two-sided Wilcoxon signed-rank test, P = 0.73). Source data are provided as a Source Data file.

Supplementary Tables

Lighting x reference-frame ANOVA on the mean vector length of polar firing rate histograms

**Factor**	**Sum square**	**Degrees of freedom**	***F***	***P***
light	1.39	1	14.8507	1.19×10^{-4} ***
reference-frame	46.46	2	247.4901	4.95×10^{-99} ****
light * reference-frame	0.35	2	1.9007	1.49×10^{-1}
residual	241.519	2573		

Pairwise t-tests with corrected P-values

**Comparison**	**t**	***P***
Cardinal Vs. Bridge	0.168	8.66×10^{-1}
Lever Vs. Bridge	-12.3339	5.32×10^{-34} ****

Lever Vs. Cardinal	-12.5022	7.319×10^{-35} ****

Supplementary Table 1. ANOVA table for the data presented in Fig. 5f, together with pairwise t-test comparisons.
