# Supplementary material for: Hippocampal firing fields anchored to a moving object predict homing direction during path-integration-based behavior
Source: Nat Commun. 2023 Nov 15;14:7373. doi: 10.1038/s41467-023-42642-3 (PMC10651862; doi:10.1038/s41467-023-42642-3)
Supplement: Supplementary file 3 — Description of Additional Supplementary Files [file 41467_2023_42642_MOESM3_ESM.pdf]

File name: Supplementary Movie 1
Description: Mouse trained to press the lever of the lever box in the home base. The mouse is seen pressing the lever and collecting food rewards in the magazine. Between days, the lever changed position. The position of the lever on 3 different days is shown.

File name: Supplementary Movie 2
Description: Mouse performing three trials on the AutoPI task. One light trial and two dark trials are shown. At the end of the video, the rotation of the arena and repositioning of the lever box taking place between trials are shown.
